# HMMerThread: Detecting Remote, Functional Conserved Domains in Entire Genomes by Combining Relaxed Sequence-Database Searches with Fold Recognition

**DOI:** 10.1371/journal.pone.0017568

**Published:** 2011-03-10

**Authors:** Charles Richard Bradshaw, Vineeth Surendranath, Robert Henschel, Matthias Stefan Mueller, Bianca Hermine Habermann

**Affiliations:** 1 Bioinformatics Laboratory, Max Planck Institute of Molecular Cell Biology and Genetics, Dresden, Saxony, Germany; 2 Center for Information Services and High Performance Computing (ZIH), Technical University, Dresden, Saxony, Germany; 3 High Performance Applications, Pervasive Technology Institute, Indiana University, Bloomington, Indiana, United States of America; 4 Bioinformatics Laboratory, Scionics c/o Max Planck Institute of Molecular Cell Biology and Genetics, Dresden, Saxony, Germany; Semmelweis University, Hungary

## Abstract

Conserved domains in proteins are one of the major sources of functional information for experimental design and genome-level annotation. Though search tools for conserved domain databases such as Hidden Markov Models (HMMs) are sensitive in detecting conserved domains in proteins when they share sufficient sequence similarity, they tend to miss more divergent family members, as they lack a reliable statistical framework for the detection of low sequence similarity. We have developed a greatly improved HMMerThread algorithm that can detect remotely conserved domains in highly divergent sequences. HMMerThread combines relaxed conserved domain searches with fold recognition to eliminate false positive, sequence-based identifications. With an accuracy of 90%, our software is able to automatically predict highly divergent members of conserved domain families with an associated 3-dimensional structure. We give additional confidence to our predictions by validation across species. We have run HMMerThread searches on eight proteomes including human and present a rich resource of remotely conserved domains, which adds significantly to the functional annotation of entire proteomes. We find ∼4500 cross-species validated, remotely conserved domain predictions in the human proteome alone. As an example, we find a DNA-binding domain in the C-terminal part of the A-kinase anchor protein 10 (AKAP10), a PKA adaptor that has been implicated in cardiac arrhythmias and premature cardiac death, which upon stress likely translocates from mitochondria to the nucleus/nucleolus. Based on our prediction, we propose that with this HLH-domain, AKAP10 is involved in the transcriptional control of stress response. Further remotely conserved domains we discuss are examples from areas such as sporulation, chromosome segregation and signalling during immune response. The HMMerThread algorithm is able to automatically detect the presence of remotely conserved domains in proteins based on weak sequence similarity. Our predictions open up new avenues for biological and medical studies. Genome-wide HMMerThread domains are available at http://vm1-hmmerthread.age.mpg.de.

## Introduction

The prediction of a protein's function is one of the most valuable contributions of bioinformatics to biological research. Next to providing functional prediction for experimental design, the functional annotation of entire proteomes is nowadays a basic task of genome database providers. Among the most used resources for functional annotations are conserved domains, which are distinct structural and functional units of a protein [1]. In general, family members of conserved domains are collected and deposited in profile databases such as Pfam, SMART or CDD [2], [3], [4]. These databases can be searched by a number of different algorithms including Hidden Markov Models (HMMs) [5], RPS-BLAST [2] or Pattern Matching [6]. Although these methods work very well when sufficient sequence similarity is present, they tend to miss more divergent family members, which lie within and below the so-called twilight zone of below 20% sequence similarity. This is in many cases the result of a lack of divergent members in domain profiles resulting in profile definitions that are too strict. Consequently, in automated conserved domain searches that are applied to entire proteomes, sensitivity has to be sacrificed for the benefit of reliable predictions.

When proteins are analyzed manually, even more sensitive methods can be applied. PSI-BLAST searches [7], for instance, which use a profile of homologs as input to iterative database searches, as well as the detection of divergent superfamily- or conserved domain- members using profile-profile comparisons [8], [9] can greatly enhance the sensitivity and therefore provide new or additional information to functional predictions of individual proteins. The HHPred-server [9] as an example allows the user to build a profile of an input sequence and performs profile-profile comparisons to conserved domain databases or profile resources of fold classes like SCOP [10] or CATH [11]. HHPred works extremely well for the detection of remote sequence similarity [12], yet it has not been adapted for genome-scale searches.

The Superfamily database [13], as another example, uses sensitive profile-based searches (SAM-T99 HMM [14]) to provide structural annotation of genomes based on SCOP families. As several, overlapping profiles are used to represent a single SCOP family and SAM-T99 HMM is used that is specifically strong in detecting remote sequence similarity, this method is able to detect remote homologs to known structural families [15]. In the initial version, the main information provided was the predicted structural fold of a protein sequence. Meanwhile, extensive functional information is provided in addition (based on Gene Ontology), thereby making the database useful for functional classification of proteins based on fold classes [16].

Alternative to sequence-based searches, structural features have been recognized as useful in detecting remote sequence similarity. As the function of a protein is to a great extent defined by its structure, its fold is generally better conserved than its sequence [17], [18]. Fold recognition (also known as threading) approaches have proven most successful in this area [19], [20]. In most fold recognition applications, a protein sequence is compared to a set of three-dimensional protein structures and is scored based on statistical approaches [17], [18], [21], [22], [23]. Though fold recognition techniques are constantly improving, it is nevertheless still difficult to reliably score a threading hit. For many approaches, one sequence can produce multiple threading hits that are hard to distinguish by score alone [24]. One common approach is to include information from other sources like putative biological function to reliably determine true positive hits. Threading tools like 3D-PSSM [25] (now Phyre [26]), TASSER [27], which uses PROSPECTOR [28], [29] derivatives as the threading engine, or MUSTER [30] all take into account sequence-, as well as structural (secondary and/or tertiary) features when scoring hits and therefore outperform purely structure-based techniques. The SAMD method as another example, utilizes neural networks together with predicted structural properties to predict structural folds within the twilight zone [19]. However, none of the above described methods except for Threader [31] have been used systematically for genome-wide annotations.

Here we introduce the genome-wide HMMerThread resource of remotely conserved domains. Based on a much improved HMMerThread algorithm we have previously published [32], we have predicted remotely conserved domains at a proteome-wide level for eight model organisms including human. Through a combination of relaxed conserved domain database searches with subsequent fold recognition steps to eliminate false positive predictions due to high E-value settings, we provide accurate predictions of conserved domains that are well within and beyond the twilight zone of sequence similarity. We use orthology information, as well as information on key functional residues, if available, to validate remotely conserved domains. Our pipeline has achieved an accuracy of 90%, making HMMerThread an accurate application to detect remotely conserved domains. We provide genome-wide data on remotely conserved HMMerThread domains in a relational database, which is openly accessible at http://vm1-hmmerthread.age.mpg.de.

Among the remote conserved domain hits in our dataset we find a number of interesting new or additional function(s) that could be assigned to proteins associated with a selected number of biological processes and human diseases. Our data allow for many predictions that can be functionally tested and thus open up completely new avenues in experimental research. In conclusion, with the HMMerThread database we have created a rich and accurate resource of remotely conserved domains of great value to experimental biological and medical research.

## Results

### Major modifications and improvements of the new HMMerThread software

**Figure 1:**
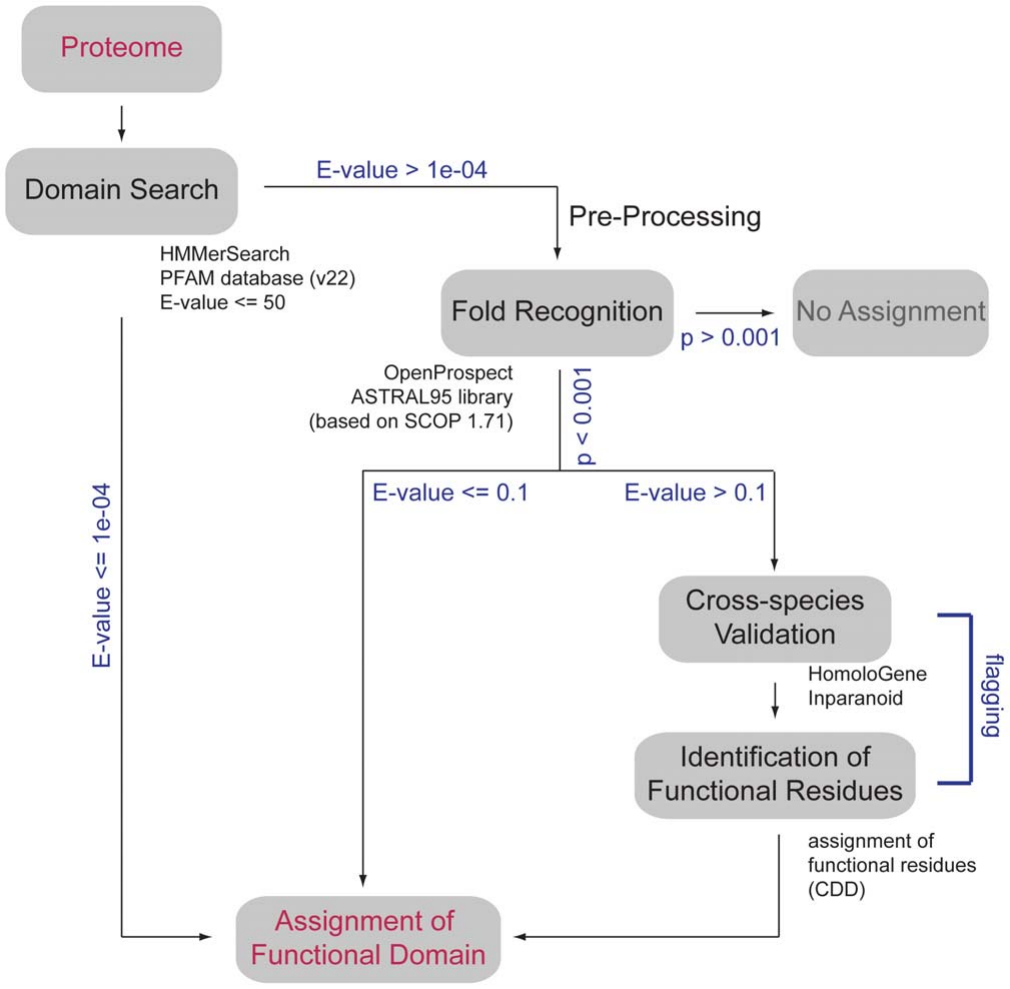
Architecture of genome-wide HMMerThread searches. Each protein of a species' proteome is sent to a conserved domain search using HMMER2 against the Pfam database with an E-value threshold of 50. If a conserved domain with an E-value below 1e-04 is detected, it is positively scored. In case an identified domain has an E-value above 1e-04, a pre-processing and fold recognition step is performed. In case of a positive identification (p<0.001), a conserved domain is scored, if the HMMER2 E-value of the conserved domain is below 0.1. If the HMMER2 E-value is above 0.1 and the associated fold has been scored positively, a cross-species validation is performed and essential residues are flagged for a confident assignment of a conserved domain.

The HMMerThread software searches for remotely conserved domains in proteins by a combination of relaxed sequence-based conserved domain searches with a subsequent fold recognition step to eliminate false positive domain hits resulting from high E-value thresholds [32] (Figure 1). We have adapted the HMMerThread algorithm to handle entire proteomes. For genome-wide annotations, conserved domain searches were carried out using the HMMER2 software (version 2.3.2) [5] with an E-value threshold of 50, which allows for the detection of sequence relationships well beyond statistically significant thresholds. A single HMMerThread run works as follows: first, an HMMER2-search is run against the Pfam database. In case a conserved domain with an E-value above the significance threshold of 1e-04 is detected, a subsequent fold recognition step is carried out. Provided that the structure(s) of the expected conserved domain is (are) positively identified, the conserved domain is scored as a positive hit. If the E-value of the conserved domain search is greater than 0.1, a validation procedure is carried out to ensure correct identification of a weak conserved domain hit. Validation steps include the identification of the same conserved domain in at least two of the orthologs of 3 related species, if available (see Supplemental Table S1), as well as identification of essential functional residues provided by the CD database [2].

#### Improvement of the threading module

We needed to take two factors into consideration, when choosing a threading engine for genome-wide HMMerThread searches: First, we needed an algorithm, which was parallelizable and readily adaptable for a high-performance computing setting. Second, we wanted to ensure good performance of the threading engine. Among the tools available, only few algorithms fulfilled the first criterion. Next to Threader3.5 [22] we have used previously, OpenProspect [33] was readily useable and adjustable for the available high-performance computing setting. We chose to use it for genome-wide HMMerThread searches, as it outperformed Threader3.5 in our tests (data not shown). Searches were furthermore carried out against the ASTRAL structural library [34].

#### Improved scoring function of the fold recognition module in HMMerThread

Our attempt on unsupervised fold recognition required the development of a reliable scoring of a threading hit. The Z-score of threaded structures in OpenProspect cannot be easily interpreted and very diverse structure families can give similar Z-scores for the same sequence. Yet, as our approach combines sequence similarity searches together with fold recognition, we could take advantage of knowing *a priori,* which structural folds to look for. Therefore, we considered two factors for scoring hits from fold recognition in HMMerThread:

1. We considered the Z-score from OpenProspect, as it represents a guide to the strength of a hit. Given a certain Z-score, we wanted to deduce a p-value reflecting the probability that this Z-score can be treated as significant. We therefore constructed a cumulative distribution function of the Z-scores for all structures in SCOP for a random set of 1000 human proteins. This gave us a naïve probability for each possible Z-score. The threshold at 0.5% of all threaded structures (∼top 60 hits) was determined as stringent after examining results of test sets containing known domains (E-values<0.0001) and remotely conserved domains.
2. We considered the number of structures associated with a conserved domain that were positively identified by OpenProspect. As many conserved domains have generally more than a single structural representative in the SCOP database, we used the hypergeometric probability function to determine, whether a given set of structures associated with a conserved domain was significantly overrepresented. This method enabled us to discriminate between significantly scoring structures due to their high frequency and those that are truly overrepresented and therefore a true positive hit. The top 60 hits we considered approximately equals 90% of the hits at a hypergeometric p-value threshold of 0.05 (Supplemental Figure S1).

Our final scoring scheme combines the p-value of the top representative conserved domain based on its Z-score – therefore ensuring that a structural hit is truly significant – with the p-value of the structure being seen by chance due to its representation in the structural library. For this combined probability, we determined a significance threshold of 0.001. Based on our benchmarking of the HMMerThread software discussed below, we found that this combined probability is a robust scoring mechanism for remotely conserved HMMerThread domains.

### Benchmarking of the improved HMMerThread software

We looked at two aspects of the new HMMerThread software: First, we tested the threading engine for its ability to detect fold classes. We considered this important as the threading module we used has a limited statistical framework and could also lead to a loss of true positive remote conserved domains. Second, we tested the performance of HMMerThread by calculating precision, recall and accuracy of the software.

#### Detection of conserved domains with statistically significant sequence conservation

**Figure 2:**
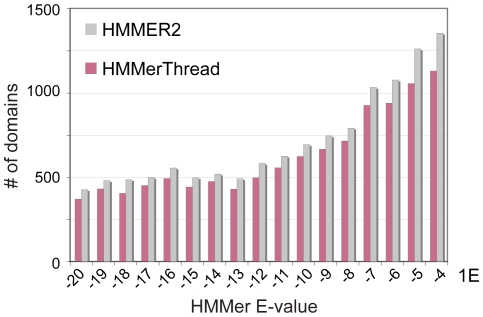
Performance of the OpenProspect software. Comparison of positive identifications of conserved domains using either HMMER2 alone (grey bars) or HMMerThread (red bars). We have tested an E-value range between 1e-20 and 1e-04 for positive identification of conserved domains by HMMerThread and 88% of conserved domains could be positively identified.

First, we analyzed the performance of the fold recognition module of HMMerThread by testing its ability to find known conserved domains with a statistically significant HMMER2 E-value. We took all detected conserved domains from the human proteome with an E-value between 1e-20 and 1e-04 and submitted them to OpenProspect for fold recognition. As shown in Figure 2, we could retrieve 88% of all known conserved domains. We also investigated whether HMMerThread could score all domain types and found that we can positively identify 60% (1920 of 3192) of domain types with OpenProspect. This is in accordance with the observation that ∼30% of domain types only occur in the prokaryotic kingdom and are not found in eukaryotes [15]. It also highlights that we cannot identify all domain types with the threading algorithm and the scoring scheme we use.

#### Calculating Precision, Recall and Accuracy of the HMMerThread software

We next set out to calculate the precision, recall and accuracy of the HMMerThread software. In order to do this, we had to identify a sufficiently high number of proteins containing remotely conserved domains that we knew were true positives. We decided to benchmark the HMMerThread software by a hide and seek procedure that involved different versions of the Pfam conserved domain database. The rationale behind this approach was that with growing domain families, more distantly related members become associated with a conserved domain profile, thereby relaxing the profile and making it more sensitive. According to this hypothesis, we would find a number of conserved domains in proteins that did not score with a significant E-value in older Pfam releases (in our case Pfam10, released in July 2003), but achieved a significant score in the newer one (Pfam22, released in July 2007 and the version used as the conserved domain database in this study). We created an overlapping dataset of the Pfam10 and Pfam22 releases, resulting in 5266 conserved domain profiles that we could test. This dataset we refer to from hereafter as the Pfam10∶22 remotely conserved domain set. We ran HMMER2 searches against both profile versions of the conserved domains using the human proteome and selected those domains, which scored with an E-value>0.1 using Pfam10, while having an E-value<0.1 in Pfam22 and where the difference between the two E-values was greater than or equal to a 10-fold change. This resulted in a total of 408 conserved domains that could be considered as true positive, weakly conserved hits in Pfam10. For these domains, we performed HMMER2 searches against Pfam10, which generated 1520 possible, overlapping hits. These, we could analyze for true positive (TP), false negative (FN), true negative (TN) and false positive (FP) identification (see Supplemental Table S2). Domain profiles that belonged to the same clan were excluded. Based on the Pfam22 profiles, 390 conserved domains were correct hits, only 18 of which we could not identify, resulting in 372 (or 95%) TPs and 5% FN. From the remaining domains, we obtained 142 (14%) FPs, which resulted in a precision of 74%.

The 14% false positive predictions with the HMMerThread algorithm is an obvious concern for automatic prediction of remotely conserved domains. In order to reduce the percentage of false positive predictions, we would have to accept too many false negative predictions (see Supplemental Figure S2 and also Supplemental Table S3). To reduce the number of false positive predictions to for instance 3%, we would only be able to retrieve 48% of true positives (see Supplemental Table S3). In order to address this problem, we wanted to know whether cross-species validation could reduce the number of false positive predictions, while retaining the good performance of the HMMerThread algorithm in recall. This also reflects the intended usage of the HMMerThread resource, where only cross-species validated domains are considered as reliable predictions. To this end, we repeated the searches with orthologs of the Pfam10∶22 dataset from mouse, dog and chicken. Validation of HMMerThread results in one species led to only a slight reduction of the false positive rate from 14% to 11%. At the same time, it reduced the number of true positive hits to 274 and increased the number of false negative predictions (note that for 79 of true positive hits, no suitable ortholog could be found for validation, which reduced the proteins that could be scored to 311 proteins with weakly conserved domains). Validation against 2 species reduced the false positive rate to 8% and the accuracy to 90%. Detection of a remotely conserved domain in all 3 orthologs pushed down the number of false positive predictions to 3%, however with a recall of only 68%. We therefore concluded that a validation against 2 species provided the best compromise in true positive and false positive predictions (see Table 1 and Supplemental Table S2). As is shown later, our dataset induced a very similar number of false positive predictions with other, very reliable algorithms like Superfamily [35]. The relatively high number of false positives therefore seems to be an inherent feature of the Pfam10∶22 dataset. In many cases, falsely identified domains contain for instance short domains like Zn-fingers, which are difficult to distinguish correctly (see Supplemental Table S2). In order to ensure low false prediction rates, we are marking domains with a length shorter than 50 amino acids in the HMMerThread database.

**Table 1:** Comparison of the new HMMerThread to its predecessor, GenThreader and the Superfamily resource.

HMMerThread (new version, 2 species validation)		HMMerThread (old version, based on Threader 3.5, ProFAT)	
True Positives	217	True Positives	186
False Negatives	47	False Negatives	197
False Positives	76	False Positives	53
True Negatives	845	True Negatives	781
False Negative Rate	18%	False Negative Rate	51%
False Positive Rate	8%	False Positive Rate	6%
*Precision*	*74%*	*Precision*	*78%*
*Accuracy*	*90%*	*Accuracy*	*79%*
*Recall*	*82%*	*Recall*	*49%*
**GenThreader**		**Superfamily**	
True Positives	121	True Positives	271
False Negatives	256	False Negatives	91
False Positives	5	False Positives	29
True Negatives	773	True Negatives	565
False Negative Rate	68%	False Negative Rate	25%
False Positive Rate	2%	False Positive Rate	5%
*Precision*	*96%*	*Precision*	*90%*
*Accuracy*	*77%*	*Accuracy*	*87%*
*Recall*	*32%*	*Recall*	*75%*

Taken together, we conclude that HMMerThread is a very powerful technique to identify true positive, remotely conserved domains and is moreover highly efficient in discriminating true positive from false positive hits.

We could confirm the good performance of HMMerThread, when we searched for remotely conserved domains that have been described in literature before and which we were well familiar with: We could confirm the existence of a BAR domain (Bin-Amphiphysin-RVS) in most of the proteins that we had previously described [36] (data not shown, please refer to BAR domains in human). We could also confirm most of the remotely conserved domains in proteins discussed in the original manuscript describing the ProFAT and HMMerThread server [32]. The RNA-Recognition-Motif (RRM_1) domain was found in the LOC84060 protein however automated HMMerThread searches without considering overlapping remote conserved domain hits did not reveal the presence of the RRM_1 domain in the Parn proteins (LOC84060 and Parn). The SAM domain (for Sterile Alpha Motif) was verified in the epidermal growth factor receptor pathway substrate 8 protein families EPS8 and EPS8L3 and we automatically detected the Acetyltransferase domain (Acetyltransf_1) in the LOC79969 proteins using the novel HMMerThread server (LOC79969). Interestingly, a large-scale screen of protein-protein interactions in worm revealed that the *C. elegans* ortholog of LOC79969, W06B11.1, interacts with a methyltransferase (C01B10.8), suggesting that this protein is part of a larger chromatin-remodelling complex [37]. We also found the previously described Acetyltransf_1 domain in the protein Eco1 from *Saccharomyces cerevisiae* [38] (Eco1). This domain was also found in the worm and fly orthologs, though we did not detect it in vertebrates or *Schizosaccharomyces pombe* (fission yeast, fly, worm, zebrafish homolog 1, zebrafish homolog 2, mouse homolog 1, mouse homolog 2, human homolog 1, human homolog 2). HMMerThread also confirmed the presence of winged-helix domains in two proteins required for meiotic recombination, Mnd1 and Hop2 [39]. Finally, HMMerThread identified a remotely conserved CARD domain in the Death Receptor 6 (DR6, aka TNFRSF21), the presence of which was also shown by structural analysis (pdb-code 2dbh, Inoue M, Koshiba S, Kigawa T, Yokoyama S, unpublished).

### Comparison of HMMerThread to its predecessor, the GenThreader and Superfamily algorithms

In order to estimate the advancement of HMMerThread in predicting remotely conserved domains compared to its previous version, as well as to other existing resources, we used the Pfam10∶22 remotely conserved domain set to calculate recall, precision and accuracy of the old HMMerThread algorithm, GenTHREADER [31] as well as the algorithm used by Superfamily [35] (see Table 1 and Supplemental Table S2).

#### Significantly improved performance of the novel HMMerThread software

When we compared the precision, accuracy and recall of the old versus new version of HMMerThread, we find that our modifications have significantly improved the software (see Table 1 and Supplemental Table S2). While we achieved a recall of 82% with the new method, we only reached 49% with the old version. The new version of HMMerThread gets a slightly worse precision with 74% versus 78% of the old version, as we have a slightly higher false positive rate (8% vs 6%, respectively). Both results are due to the very different scoring scheme of the old versus new version of the algorithm. In the old version of HMMerThread, the scoring of a remotely conserved domain consisted of identification of a positive structural hit within the first 25 identified structures. As one is to expect a slightly higher false positive rate, one also can expect a much lower false negative rate with the novel approach we have taken for scoring HMMerThread hits. Finally, the accuracy of the old versus new version of HMMerThread is 79% versus 90%. Based on these data, we conclude that we could significantly enhance the performance of the HMMerThread algorithm.

#### Comparison of the new HMMerThread to existing software for detecting remote conservation

We decided to compare the novel HMMerThread algorithm to two existing resources that provide information on remote conservation between proteins. For one, we looked at GenTHREADER [31], which uses a fold recognition pipeline to predict the putative three-dimensional structures of proteins on a genome-wide scale. In order to estimate the performance of GenTHREADER, we used the Pfam10∶22 remotely conserved domain set and applied GenTHREADER to identify the correct fold of a remotely conserved domain (for detailed description, see Methods). The performance of GenTHREADER was very comparable to the old version of HMMerThread, with a very low false positive rate (2%), but also a quite high false negative rate (68%). The recall of GenTHREADER was therefore only 32%, precision reached 96% and the overall accuracy was with 77% very similar to old version of HMMerThread (see Table 1 and Supplemental Table S2).

Superfamily [35] was the second resource we compared the HMMerThread database to. After mapping of the SCOP-IDs of a Superfamily to conserved domains of the Pfam database using PDBMAP, we chose to score a true positive hit, if a Superfamily was reported that included the correct remote conserved Pfam hit; all related Pfam families found within a SCOP family were ignored for false positive predictions, in addition to the excluded CLAN members that we used for HMMerThread or GenThreader. Superfamily was able to identify 271 of our positive conserved domain set, which resulted in a recall of 75%. It achieved a higher precision (90%), with a false positive rate of only 5%. The overall accuracy of Superfamily was 87% and therefore very similar to the new version of HMMerThread (Table 1 and Supplemental Table S2).

As noted earlier, it seems to be a built-in feature of our dataset that even precise algorithms like GenThreader or Superfamily show a higher than usual false positive rate (2% and 5%, respectively). We therefore decided that a false positive rate of 8% with our dataset was acceptable for the new HMMerThread algorithm.

We conclude from this data that HMMerThread with its unique approach to consider not only sequence-, but also structural information outperforms other methods currently available in identifying remotely conserved domains.

### Genome-wide HMMerThread Searches

We have carried out HMMerThread searches against the proteomes of the eight most common model organisms including human and detected a total of 58330 weakly conserved domains with an E-value above 0.1 (see Table 2). About 13000 of these were validated in at least one additional species and ∼6000 in two. Many of the model organisms we chose lack a third species with a reasonable phylogenetic distance. Therefore, only ∼2000 domains can be found in three other species with ∼1000 of these identified in human alone. Nearly all fold classes of the current SCOP release (1.71) could be identified using genomic HMMerThread searches, with globular domains being the vast majority of structures found (Table 3).

**Table 2:** Statistics of HMMerThread weakly conserved domains in the 8 proteomes analyzed.

Genome	Total proteins	Remotely conserved domains	3-species validation	2-species validation	1-species validation
H. sapiens	33466	12038	1031	2672	4492
M. musculus	34981	11460	636	1873	3541
D. rerio	29720	11422	-	872	1728
C. elegans	23518	7664	-	-	1741
D. melanogaster	19,388	7430	249	556	1075
S. cerevisiae	5868	1919	41	87	360
S. pombe	5004	1506	-	-	-
D. discoideum	13501	4891	-	-	-
TOTAL:	165446	58330	1957	6060	12937

**Table 3:** SCOP classes (version 1.71) identified in the 8 genome-wide HMMerThread searches.

SCOP Class	Count	Percentage
Small proteins	20297	24.46%
Alpha and beta proteins (a+b)	18250	21.99%
All beta proteins	15273	18.40%
All alpha proteins	13407	16.16%
Alpha/beta proteins (a/b)	10935	13.18%
Membrane and cell surface proteins and peptides	3185	3.84%
Peptides	677	0.82%
Multi-domain proteins (alpha and beta)	578	0.70%
Coiled coil proteins	377	0.45%
Designed proteins	7	0.01%

The results of the genome-wide HMMerThread searches are presented in a web-based relational database, which includes the results from all eight proteomes analyzed. The database can be queried by gene name, protein ID or Pfam conserved domains. The HMMerThread domains are shown along with associated annotation for the given gene from diverse sources like the NCBI, SGD, Wormbase, Flybase or HPRD. Though remote conserved domain searches were not carried out against the Pfam24 database [40], we provide Pfam24 domain annotation in the HMMerThread database. We show the HMMerThread domains graphically in direct relation to conserved domains from InterProScan [41], which gives the user an immediate overview of the presumed functions of the protein under study. Next to the Pfam domains, we integrate PROSITE sequence-based features from pattern matches underneath the domain. Remotely conserved HMMerThread domains are colour-coded based on their validation status (green  =  no validation required (HMMER2 E-value<0.1), red  =  present in 3 validation species, orange  =  present in 2 validation species and yellow  =  present in 1 validation species, grey  =  no validation data available). The validation information is furthermore provided by a mouse-over popup on the domain images (Supplemental Figure S3). We extract species-specific information for further annotation of entries; records from human for instance contain NCBI gene summaries, GO terms and HPRD interactions, while entries from *S. cerevisiae* contain summaries and GO terms from SGD. For human records, we integrate iPfam data along with domains that may explain interactions in a separate table. We also provide a “live search” feature, where sequences that have not been processed by us can be searched using the HMMerThread pipeline with an updated version of the Astral database (the currently used version is 1.73). The database is publicly available at http://vm1-hmmerthread.age.mpg.de.

### Novel functional predictions based on remotely conserved domains

We set out to search for novel functional predictions based on detected HMMerThread domains. To do this, we followed several strategies: 1) we searched for undiscovered, remotely conserved domains in proteins that were detected in genome-wide functional screens. We discuss the overall statistics of a genome-wide functional dataset on factors involved in Hepatitis C Virus replication in human cells and describe one more detailed example with potential mechanistic insight. 2) We looked for remotely conserved domains in proteins associated with a biological process. We focused on mitotic and meiotic genes, as well as genes associated with diseases using the OMIM resource (NCBI) [42]. We present two examples for each category. 3) We searched for domains within domains, as weak functionally conserved domains with a known function are often found within DUF (Domain of Unknown Function) domains. 4) We looked at domain-domain interaction data that might shed light on the binding sites and mode of interaction between proteins.

### Remotely conserved domains in proteins identified in functional screens

We have chosen a functional screen for cofactors of Hepatitis C Virus replication in human cells [43], whose hits we have annotated using HMMerThread in addition to InterProScan domains. Among the genes that were involved in viral replication, those associated with Golgi vesicle binding, organization and biogenesis were overrepresented (see Supplemental Table S4). In this dataset, 29 remotely conserved HMMerThread domains are found, which are predominately involved in protein binding activities.

#### The transcriptional repressor Nab1 contains a remotely conserved SAM domain

**Figure 3:**
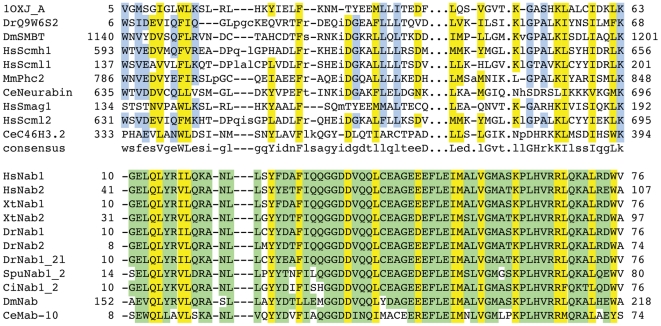
Multiple sequence alignments of remotely conserved domains in proteins identified in functional screens. Multiple sequence alignment of the Nab1 family with the SAM domain family (taken from CDD). Residues that are conserved between the two families are highlighted in yellow, those found in only one of them are highlighted in blue and green, respectively. Essential, functional residues retrieved from the CD database are indicated by hash keys. Accession numbers of sequences can be found in Supplemental Table S7.

Among the hits that showed significant reduction of viral replication with more than two independent silencing triggers was the gene Nab1 (NGFI-A binding protein 1). Nab1 is a transcriptional co-repressor that interacts directly with early growth response transcription factor 1 (Erg1) and thereby either positively or negatively modulates transcriptional activation of early response genes [44], [45]. Egr1 itself has been implicated in Hepatitis Virus C infection through the activation of IGF-II (insulin growth factor II) gene expression, which is a critical factor during the formation of hepatocellular carinoma (HCC) [46]. The fact that Nab1, a stable interactor and transcriptional co-factor of Erg1 is found in a screen for viral replication of Heptatitis C virus raises the possibility that Nab1/Erg1 is already actively assisting Hepatitis C pathogenesis by helping viral reproduction. Moreover, it opens the possibility that not only transcriptional activation, but also repression via Erg1 might be required for efficient replication of the virus. This is in accordance with the observation that Hepatitis C virus not only induces, but also represses the transcription of a set of genes [47], [48]. We detected an N-terminal SAM (Sterile Alpha Motif) domain in the Nab1 proteins, which lies within their N-terminal NCD1 domain (Figure 3). The SAM domain of Nab1 shows sufficient sequence conservation for detection by PSI-BLAST searches [7] (data not shown). SAM domains are thought to be protein interaction domains and are found in a number of proteins that are involved in different developmental processes throughout the eukaryotic kingdom [49]. The simplest functional implication of the presence of a SAM domain in Nab1 is that this domain provides the interaction interface to Egr-1. However, SAM domains among others are also found in transcriptional repressors such as the TEL protein. Transcriptional silencing by TEL has been proposed to involve oligomerization of its N-terminal SAM domain, building a proteinaceous core around which the DNA is wrapped, thereby enabling spreading of the repressor activity [50]. Although it is not clear, whether the SAM domain of Nab1 is capable of oligomerization, the presence of this conserved SAM domain in Nab1 suggests that Nab1 plays an essential role during Hepatitis C-induced transcriptional repression or activation.

### Prediction of molecular mechanistic function to generate testable hypotheses for proteins involved in cell division and proteins associated with human diseases

In order to provide examples of the predictive power of the HMMerThread method, we set out to search for remotely conserved domains in proteins involved in mitosis and/or meiosis, as well as genes associated with human diseases (taken from OMIM) that could elucidate their molecular mechanism.

#### Yeast Ssp2 harbours a RNA-binding domain and might be involved in mRNA localization during sporulation

**Figure 4 pone-0017568-g004:**
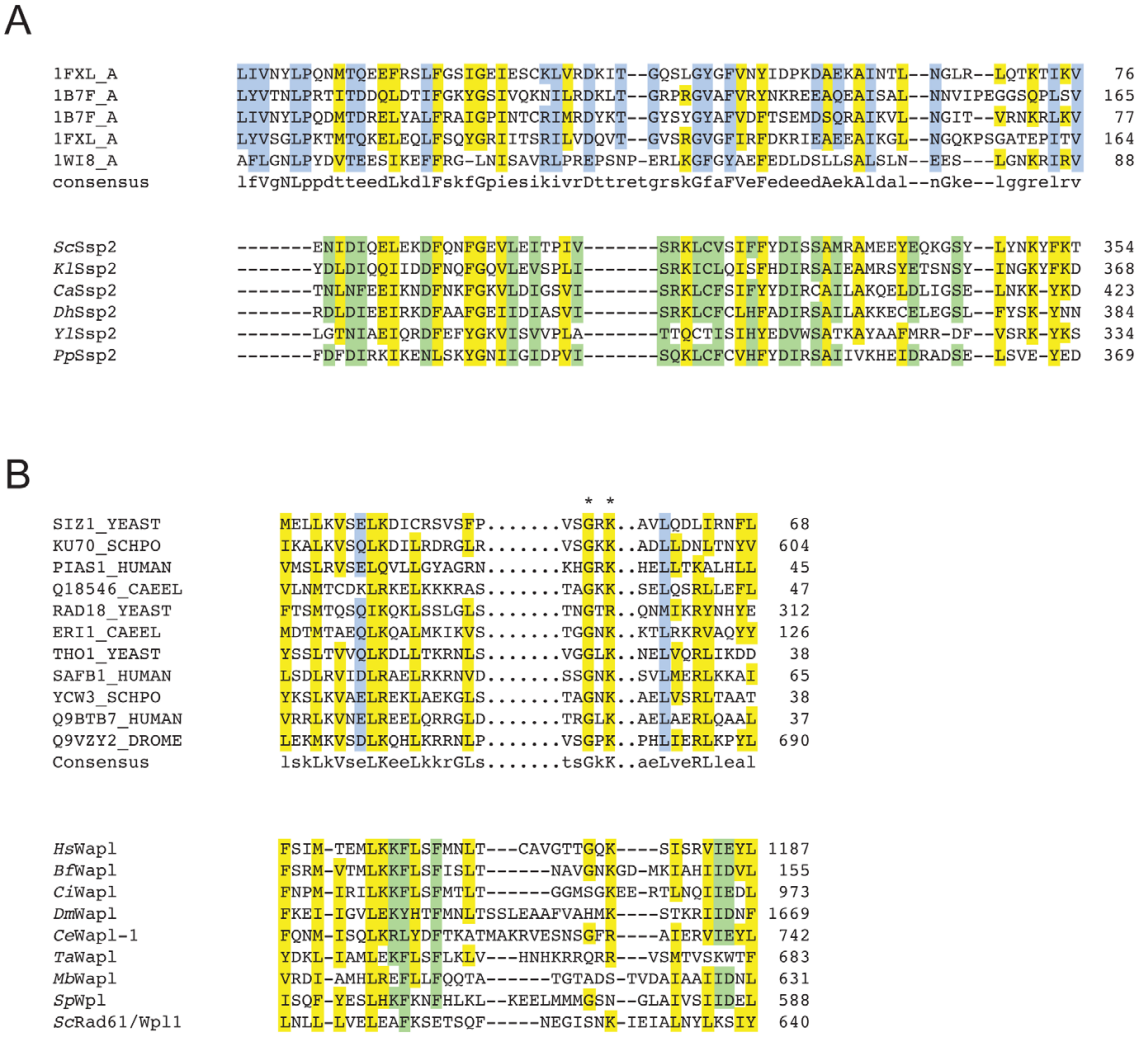
Multiple sequence alignments of remotely conserved domains in proteins associated with mitosis and meiosis. (**A**) Multiple sequence alignment of the Ssp2 family with the RRM_1 domain. (**B**) Multipe sequence alignment of the Wapl/Rad61 family with the SAP domain family. Residues that are conserved between the two families are highlighted in yellow, those found in only one of them are highlighted in blue and green, respectively. Essential, functional residues retrieved from the CD database are indicated by hash keys, those retrieved from literature (SAP domain) with stars. Accession numbers of sequences can be found in Supplemental Table S7.

Among the weak, conserved domain hits was an RRM_1 domain we found in Ssp2. This protein is required for the proper formation of the prospore membrane (PSM) and the spore wall (SW) during sporulation (Figure 4 A). We could confirm this remotely conserved domain by PSI-BLAST [7] searches (data not shown). Yet, why is a RNA-binding domain found in a protein involved in spore wall formation? Ssp2 is specifically required for vesicle fusion during formation of the PSM, as the *ssp2* null mutant can be partially rescued by overexpression of proteins from the vesicle fusion machinery, namely the phospholipase D Spo14, and the t-SNARE protein required for meiosis, Sso1. At least Spo14, together with Ssp2 is specifically localized to the PSM after the second meiotic division. Interestingly, when Oyen and colleagues [51] analyzed the sporulation-specific functions of Sso1, they found that next to functional domains within the protein itself, the 3′UTR of the sso1 mRNA is essential for sporulation and this function cannot be rescued by the 3′UTR of its close paralogue, sso2, which does not have a meiosis-specific function. In the same report, Oyen et al. tested for expression levels of sso1 and the sso2 paralogue, and found no difference between the two genes, which suggests that translational control does not play a role in the sporulation-specific function of the sso1 3′UTR. This raises the possibility that proper localization of sso1 mRNA is essential for the function of the Sso1 protein in late meiotic events, suggesting that potentially mRNA localization plays a crucial role in sporulation. The RNA-binding protein Ssp2 might therefore be essential for the proper localization of the mRNA of one – or multiple – genes during late meiotic stages to the PSM.

#### A putative SAP domain was found in the C-terminus of the WAPL proteins

The Wapl (*Wings-apart like*) protein was first described as a heterochromatin organizer in fly, whose loss leads to chromosome missegregation in meiosis [52]. Subsequently, Wapl was identified as an essential player in chromosome segregation in mitosis, as it is physically associated with two cohesin subunits (Pds5 and Scc3) and associates with DNA at the same location as cohesin [53]. The cohesin complex is a ring-like structure that entraps sister chromatids and thereby ensures the accurate segregation of chromosomes at the metaphase to anaphase transition and enables efficient repair of DNA double-strand breaks (DBS) in G2 [54], [55]. In vertebrate cells, Wapl is required to remove cohesin from chromosome arms during prophase and prometaphase and promotes rapid turnover of cohesin during interphase. Loss of Wapl leads to ‘hypercohesed’ mitotic chromosomes [53], [56]. Loss of the yeast ortholog of Wapl, Rad61/Wpl1, in contrast, results in weak impairment of cohesion [57], [58], which led the authors to speculate that the Pds5/Scc3/Wpl1 complex helps in the maintenance of cohesion ring closure around DNA [57]. In order to reconcile the two opposing functions of Wpl1/Wapl in vertebrate and yeast cells, Peters [59] proposed that the Wapl proteins perform multiple roles in DNA-cohesion interaction, cohesion establishment, maintenance and dissociation and that depending on the cellular system, one or the other function of Wapl will display a phenotype. So far, it is not understood how Wapl enables either the stabilization of the cohesin ring around DNA molecules or how it promotes loss of cohesion during prophase. It is also not known, which other functions Wapl might fulfil. We found a C-terminal SAP domain in the vertebrate Wapl proteins (Figure 4 B). SAP domains occur in a multitude of DNA binding proteins that contain a diverse set of other functional conserved domains. They bind to AT-rich chromosomal regions and were first described in chromosomal organization [60]. We propose that the predicted SAP domain of Wapl could be directly associated with chromosomal DNA. The SAP domain, being very short and if a true positive, degenerate in Wapl proteins, can in this case not be verified using sequence-based methods. When threading this region using Phyre [26], however the majority of the identified structures are DNA-binding domains with a helix-loop-helix topology. The highly conserved Lysine residue in the loop region of the two helices, as well as the conserved positive charge in the first helix are present in most, yet not all Wapl orthologs. The putative SAP domain of the yeast Rad61 protein shows for instance low conservation on sequence level and some of the phenotypic differences between species upon loss of this protein might be explained by this observation.

#### A putative CUT-like Helix-Loop-Helix (HLH) domain was found in the C-terminus of AKAP10

**Figure 5 pone-0017568-g005:**
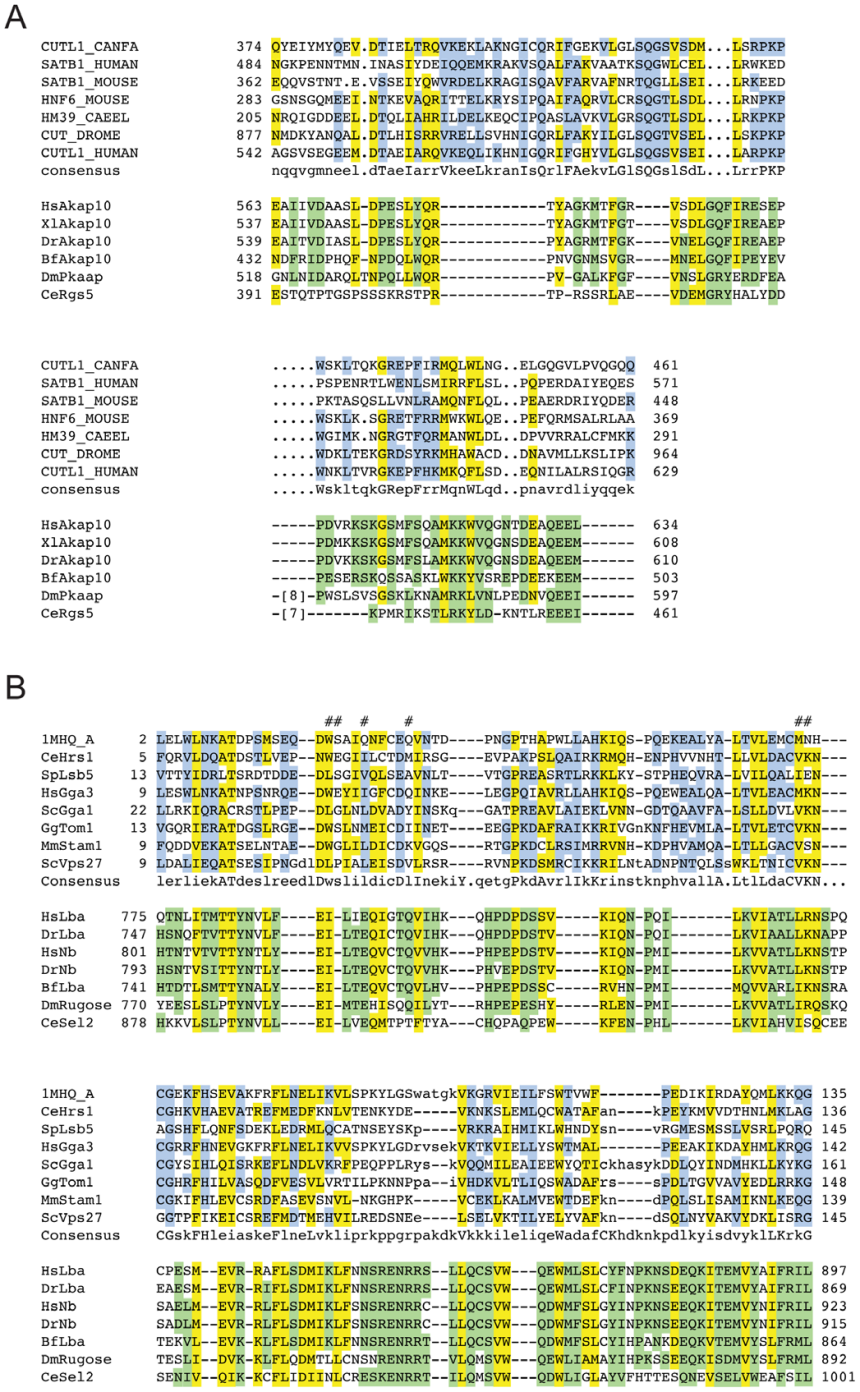
Multiple sequence alignments of remotely conserved domains found in proteins associated with human diseases. (**A**) Multiple sequence alignment of the AKAP10 family with the CUT-like HLH domain family. (**B**) Multiple sequence alignment of the Lba protein family with the VHS domain family. Residues that are conserved between the two families are highlighted in yellow, those found in only one of them are highlighted in blue and green, respectively. Essential, functional residues retrieved from the CD database are indicated by hash keys. Accession numbers of sequences can be found in Supplemental Table S7.

The dual-specific A-kinase anchor protein 10 (AKAP10) is member of a diverse protein family, which binds to the regulatory subunit of protein kinase A (PKA, for a review on AKAP10, see [61]). Unlike other AKAP family members, it contains two central RGS (Regulator of G-protein Signaling) domains, which are usually found in GTPase activating proteins (GAPs) for G-proteins [62]. The RGS domains in AKAP10 interact with the recycling small GTPases Rab4 and Rab11 [63], making this protein a switch point between signalling and endocytosis. The protein is expressed in all tissues and seems to be enriched in mitochondria [64]. An Isoleucine to Valine mutation in the C-terminal PKA interacting motif that leads to three-fold higher affinity for PKA, has been associated with higher mortality [65]. Humans carrying this mutation show an increased basal heart rate and decreased heart rate variability. Mice carrying the same allele show cardiac arrhythmias and die prematurely [66], which suggests that AKAP10 plays an essential role in the control of heart rhythm and which makes it an interesting medical target. We found a C-terminal CUT-like HLH-domain in the AKAP-10 proteins of human and mouse (Figure 5 A) right adjacent to the PKA interacting motif. CUT domains are DNA-binding domains that either bind alone or in combination with homeodomains, many of which are actually found in the same protein [67]. The weakly conserved HLH like fold (the HMMER2 E-value is 6.9) can again be verified by PSI-BLAST searches, which identify HLH domain proteins like microphthalmia-associated transcription factor (for instance the human protein NP_006713) and transcription factors EB from diverse species. Interestingly, no true CUT domain can be found by PSI-BLAST searches (not shown), which opens the possibility that the putative DNA-binding domain of AKAP10 might be member of a different HLH family. Yet, what function does a DNA-binding domain perform in a protein predominantly localized to mitochondria and presumably involved in signal transduction and recycling? It has been shown that AKAP10, together with other proteins carrying a RGS domain undergoes nuclear/nucleolar translocation upon mild heat, proteotoxic stress or the overexpression of Heat Shock Transcription Factor 1 (HSF1). Some members of the RGS-domain family that also carry winged-helix DNA binding domains are thought to be involved in stress-induced gene expression (see [64] and references therein) and it is possible that AKAP10 either alone or in combination with another homeodomain transcription factor is involved in regulating stress-related gene expression. AKAP10 therefore might again represent a multifaceted switch-point in the cell that combines signalling, recycling and transcriptional response.

#### A VHS domain in the chs1/beige protein Lba is implicated in immune deficiency

The maturation of B-cells, monocytes and dendritic cells is induced by Lipopolysaccharide (LPS) that stimulates the response of the cells to bacterial pathogens. The protein Lba (for LPS-responsive, beige-like anchor gene, aka Lrba) has been identified as a gene that is expressed in response to LPS and is involved in the maturation of immune cells [68]. Lba has also been associated with Chediak-Higashi syndrome (CHS), which is characterized by a severe immune defect among other symptoms [68]. Like other members of the *chs1/beige* family, mutations in Lba lead to perturbed intracellular trafficking and it seems that Lba function is essential for polarized transport of intracellular trafficking. Lba also carries features of AKAP (A kinase anchor proteins), namely the ability to bind to Protein kinase A (PKA). The Lba-GFP fusion protein translocates from cytoplasm to intracellular vesicles upon stimulation with LPS and can be found on Golgi membranes, lysosomes, ER, plasma membrane and endocytic vesicles [68]. It is so far unknown, which domain is required for the association of Lba with membranes. *In vitro* experiments precluded binding of the PH-BEACH domain to phospholipids [69]. We discovered a VHS-like domain in the N-terminal region of the Lba proteins (Figure 5 B). Though sequence similarity is very weak, we can verify the VHS domain by profile-profile comparisons (hhpred, [9]) and fold recognition (Phyre, [26]) (data not shown). VHS domains have been implicated in cargo recognition and vesicle trafficking [70] and are found in a variety of proteins involved in intracellular transport [71]. When found in GGA (Golgi-localized, γ-ear containing, ARF-binding) proteins, the VHS domain binds to a subset of sorting receptors that move and transfer cargo between the trans-golgi network (TGN) and the endosomal compartment [72]; [73]; [74]; [75]. Though VHS domains occur in combination with different accessory conserved domains, their molecular function is presumably identical. Interestingly, they are mostly found in the very N-terminal regions of the proteins and this localization is thought to contribute to their function, though no experimental proof for this hypothesis exists [70]. As the predicted VHS domain of Lba is not in the very N-terminus of the protein, we propose that this domain is VHS-like and might have at least a subset of similar functionality to the N-terminal VHS domains in cargo sorting required for polarized membrane trafficking. It is furthermore possible that splice variants of the full-length Lba protein lacking the N-terminal part might exist that thus harbour the VHS domain in their very N-terminus. Moreover, the presence of the PKA interacting motif in the Lba family indicates that these proteins serve as linkers between signalling and trafficking. Via the proposed VHS domain, they might ensure proper channelling of extracellular stimuli within the intracellular membrane network.

### Novel, weakly conserved domains within conserved domains of unknown function (DUF)

Domains of unknown function (DUF) are often annotated in case a clear block of sequence similarity is found within a protein family with unknown function. Domain profiles of DUF-domains are often restrictive, as not many members have been assigned to these conserved domain families. When analyzing the data from HMMerThread searches, we frequently found remotely conserved domains of known function within DUF-domains. For example, a Calponin Homology (CH-) domain lies within the N-terminal region of the DUF1042 domain (see for instance the Spef1 protein in human, mouse or fish). Other examples are methyltransferase_11 domains hidden in the DUF689 domains (DrXP_684963 or DrCiapin1), which are easily verifiable by PSI-BLAST searching and the DUF738 domain, which contains an Acetyltransferase domain (Acetyltransf_1) that was already described in an earlier section of this manuscript (see for instance the LOC79969 proteins from human, mouse, fish or worm). A complete list of conserved domains with an associated function within DUF domains can be found in Supplemental Table S5.

### Identification of potential interaction sites and prediction of interactors due to novel, weakly conserved domains

Interaction between proteins often takes place via conserved domains and this type of data is stored in the iPfam database [76]. We used remotely conserved HMMerThread domains in the human proteome to search for interaction sites of previously known interaction partners that we have extracted from the HPRD resource [77]. The presence of a remotely conserved domain can also reveal potential interactors that have so far not been predicted. A full list of known interactors and their interacting domains based on remotely conserved domains can be found in Supplemental Table S6.

Among the HMMerThread hits we found in the human interactome was a weakly conserved RPH3A_effector domain (HMMER2 E-value of 11) in the protein exophilin 5 (Exph5, aka Slac-2), which overlaps with the PROSITE Rab-binding pattern. The RPH3A_effector domain was initially described as a Rab3 interaction motif found in the Rabphilin-3A protein [78] and is structurally related to the Slp homology domain (SHD). Slac-2/Exph5 uses this domain to interact specifically with Rab27A [79]. HMMerThread could successfully detect this weak sequence relationship.

**Figure 6:**
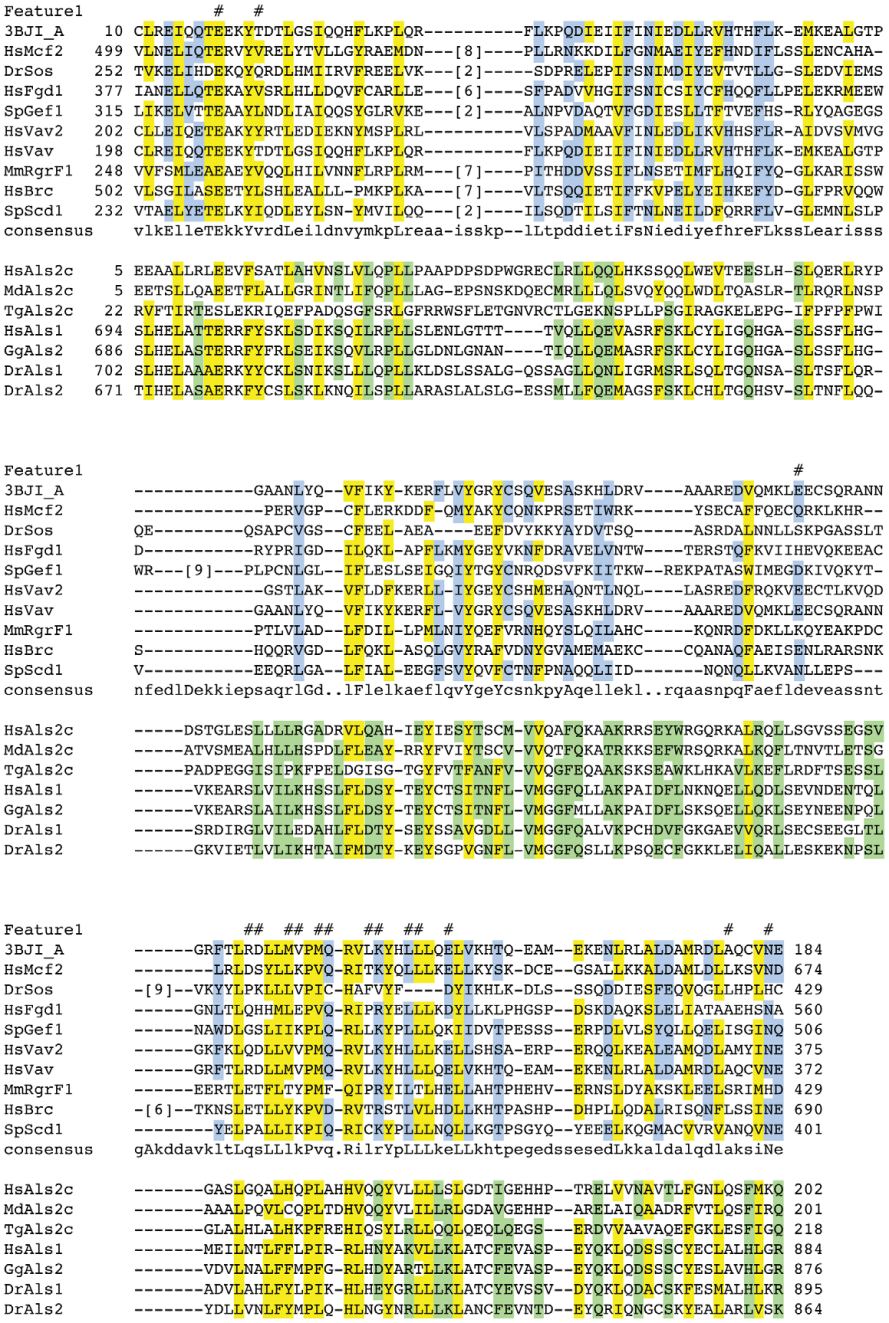
Multiple sequence alignment of the Als2cL family with the RhoGEF domain family. Residues that are conserved between the two families are highlighted in yellow, those found in only one of them are highlighted in blue and green, respectively. Essential, functional residues retrieved from the CD database are indicated by hash keys. Accession numbers of sequences can be found in Supplemental Table S7.

Another remotely conserved HMMerThread domain is the RhoGEF/DH domain of the protein Als2cL, a closely related protein to Alsin (Als2), which lacks the N-terminal RCC (regulator of chromosome condensation) domain [80], [81] (Figure 6). Like Als2, Als2cL has a C-terminal VPS9 domain, which acts as a GEF for Rab5a. The protein was shown to form homodimers, which are able to interact with Als2 oligomers and these complexes localize to vesicular structures within the cell. Though Als2cL and Als2 share extensive sequence similarity over large parts of their sequence, their molecular functions seem distinct. Als2cL, for instance negatively modulates the endosome enlargment phenotype observed in Als2 mutants that have constitutive Rab5 GEF activity and rather leads to tubulation of endosomal compartments [80]. Next to its function in endosomal compartment dynamics, Als2 also regulates Rac-PAK signalling in neurite outgrowth [82] and it does so by acting as a GEF for Rac via its central RhoGEF domain. So far, binding to a Rho-type GTPase like Rac has not been reported for Als2cL. Furthermore, though the presence of the RhoGEF and PH domain in the N-terminus of the protein has been stated [81] and though the presence of this domain can be verified using PSI-BLAST or BLAST searches [7] (data not shown), this domain is not detected via standard domain search programs as it has an E-value of 4. We propose that like Als2, Als2cL will also interact with and act as a RhoGEF for a Rho-type GTPase, as there are few amino acid exchanges between Als2 and Als2cL in the essential residues.

## Discussion

With the HMMerThread method we attempt to provide conserved domain predictions beyond the statistical threshold of purely sequence-based methods. By relaxing significance thresholds of sequence-based conserved domain searches and selecting for true positive hits by subsequent fold recognition, we can go far into and beyond the twilight zone of sequence similarity to detect remotely conserved domain members. We can significantly improve the precision of weak conserved domain predictions by cross-species validation of HMMerThread hits. The new implementation of HMMerThread shows a clearly superior performance to our previously published version of the software. We have raised the accuracy of our predictions from 79% to 90% and in this surpass existing methods of genome-scale detection of remote conservation between proteins that are either based on sequence, or structure alone. We could positively identify a number of remotely conserved domains previously reported in literature. We have discussed a number of highly interesting examples of weak, conserved domain hits that are associated with specific functional screens, cellular processes or human diseases. Our predictions could explain in part the observed phenotypes and open up new avenues for experimental studies. In this, the HMMerThread resource provides a rich resource of sensitive, functional annotations of proteins for all major model organisms. In the human proteome, we found ∼12000 remotely conserved domains with an E-value above 0.1. Of those ∼4500 could be validated in at least one other species and ∼2700 were validated against 2 species. This data enhances greatly the ability to functionally characterize many proteins and demonstrates that our knowledge of protein functions can be increased based on more sensitive searches against the current databases.

One improvement to the previous version of HMMerThread was the implementation of a reliable scoring scheme for HMMerThread hits so that the software could be applied to entire genomes without manual interference. In contrast, the Z-score of OpenProspect does not effectively discriminate between true- and false- positives and the confidence measure the authors describe for Prospect II [33] is not incorporated in the available OpenProspect software version. For the new scoring approach in HMMerThread, we have taken into consideration not only the Z-score of the threading run, but also the number of structures positively identified within a given Z-score threshold. Our data suggests that this combined p-value has strong discriminative power to distinguish between false positive and true positive hits. However, the strength of the p-value we derive from the hypergeometric distribution and therefore also of the combined p-value depends on the number of structures associated with a conserved domain.

In order to improve the quality and the reliability of our conserved domain predictions, we chose to validate remotely conserved domains by confirming their presence in the orthologs of other, related species. We found that this is a very good measure for the reliability of weak conserved domain predictions and employed this strategy when using HMMerThread domains for annotating proteins in genome-wide screens. This procedure however is highly dependent on a) the availability of the complete and annotated genome of at least one related species and b) the quality of the genome annotations. We do not try to predict genes in genomic sequences and are relying on the predicted CDS provided by genome databases. Clearly not all genes are correctly predicted, if predicted at all, in less common model organisms such as the mosquito *Anopheles gambiae*, *Fugu rubripes* or chicken. Moreover, we do not find any usable proteome information for the close relatives of some of the chosen model organisms, like *Schizosaccharomyces pombe* or *Dictyostelium discoideum*. In these cases, we do not have any validation information based on orthologous sequences. These problems will however be solved in the future, as more genomes are being sequenced and as the annotation status of genomes from non-model organisms improves over time.

A second approach we take is to validate remotely conserved domains by looking for the presence of essential residues provided by the CDD resource [2]. This method is often restricted by a lack of annotation and - in many cases – by the lack of knowledge on functionally critical residues. This verification step however, can be of high value, as it can be used to discriminate between a certain sequence just adopting a particular fold rather than actually fulfilling the associated function(s).

Contrary to purely structure-based techniques, HMMerThread can only detect remotely conserved domains, whose structure has been solved. Given this fact, we limit ourselves to conserved domains that have an associated three-dimensional structure. We currently can cover about 35% of the conserved domain sequence space. With newly solved three-dimensional structures, we can update our database with low effort, as we can specifically look for newly added structures. Due to the much smaller database sizes, we can therefore greatly reduce the required processing time for updates.

Another limitation we have chosen to accept is to ignore overlapping conserved domains and limit the fold recognition step to the top hit of the HMMER2 search. This was purely due to limitations in computational resources. Based on statistics from the yeast proteome, in which we have attempted to discriminate the true positive conserved domain hit in a set of overlapping domains, we estimate that we miss roughly 54% true positive hits in the other organisms, which we could only retrieve through an eight-fold increase in run-time. This data again demonstrates the power of our approach, as in more than 50% of the cases, the true positive hit is not the first one that is detected in the sequence-based search. We are currently working on an updated version of the database that includes overlaps in all organisms presented. Finally, updating of the HMMerThread database with novel software releases will result in the highest cost concerning computing time. Meanwhile, the Pfam24 database has been released and we have included this data in our resource. Live searches using HMMerThread already use the new release of the Pfam database. We will furthermore incorporate future releases of Pfam in updates of the HMMerThread database and we will do the same for the fold library, SCOP. Likewise, we will use HMMER3 for updates, once it is out of beta testing.

Remotely conserved HMMerThread domains in the HMMerThread resource are a valuable guideline for further experimental studies of protein function. Often, a remotely conserved HMMerThread domain is the sole information available for a protein under study and it provides clues for experimental design to elucidate the mechanistic function of a protein. Moreover, HMMerThread has demonstrated high precision, recall and accuracy. Yet, it is clear that conserved domain prediction based on weak sequence similarity is essentially a prediction and will need further verification. Moreover, as we partly rely on fold recognition, HMMerThread predictions have to be considered as clan-based predictions of conserved domains. All remotely conserved domains that are discussed in this manuscript have been verified by independent methods like PSI-BLAST, profile-profile comparisons or pure fold recognition using algorithms other than Prospect II/OpenProspect. We suggest, when analyzing proteins in low throughput, to use remotely conserved HMMerThread domains as a starting point for functional prediction and – especially when looking at remotely conserved domains with very low sequence similarity – to proceed with an independent verification step.

We are currently working on a downloadable version of the HMMerThread tool. Provided that validation data can be generated from a related species, HMMerThread will prove to be a highly useful approach for sensitive conserved domain annotation in entire genomes.

## Materials and Methods

### HMMerThread application

HMMerThread was implemented using Pfam Release 22 and SCOP release 1.71. The pipeline was implemented in 4 components and in the Perl 5.8 scripting language without dependencies to allow for execution on various HPC platforms. The 4 components include domain searches (HMMER2.3.2, [5]), pre-processing (PSIPred secondary structure prediction [22], SEG detection of low complexity [83], NCoils detection of coilded-coil regions [84]), threading (OpenProspect [33]), post-processing (scoring).

### Domain search

The first step of the HMMerThread pipeline is to search for Pfam domains in the full-length sequence. Genome-wide runs were done using an HMMER2 in global search mode and with an expect value threshold of 50. Once identified domains were extracted, they were ranked according to their e-values. Overlapping domains were removed leaving only the best scoring domains for each region of the sequence. For all conserved domains with score higher than 1e-04, the PDBMAP was consulted to see if a structure exists for the given conserved domain. If a structure was present, the domain was sent to pre-processing.

### Pre-Processing

For pre-processing, secondary structure prediction with PSI-Pred was performed on the entire protein sequence. After this, SEG and NCoils were run to remove regions of low complexity and coiled coils from the input sequence. Data from these 3 programs were collated into a single sequence (with “X” for regions of low complexity and coiled coils) and the domains from the domain search step were retrieved from of the pre-processed sequence to be sent for threading.

### Threading

Threading was performed with OpenProspect on the input sequences from pre-processing. Searches were done on a high-performance computing (HPC) system. Settings included the use of “full” Z-scores (option -zscore_full) and 100 Z-cycles (option -zcycles 100). Runtime for an input file varied in accordance with the sequence length. The average runtime was ∼ 4 hours on a single core of a 2.6 GHz AMD x85 Opteron processor. HPC was provided by the ZIH (TU-Dresden) in the form of a PC Farm of 2,584 cores. The processing of all 8 species took ∼3,000,000 CPU hours including cross-species validation.

### Post-processing

Post-Processing was performed in 2 steps. Firstly, the results of the Threading run were processed. This involved extracting the key parameters (Z-Score and position) from the output file. These parameters were ranked producing a hit list for all SCOP domains (12,430 domains for SCOP 1.71). Secondly, scoring was performed on this ordered list based on two factors. The first was on the p-value from the naïve probability generated from a cumulative distribution function (CDF) of the Z-Scores from 1,000 OpenProspect runs (∼12 million Z-Scores) and the second is based on the cumulative probability from a hypergeometric distribution. Therefore:
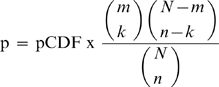



where pCDF is the p-value of the Z-Score from the scoring of expected structure, N  =  all structures threaded, n  =  top 60 structures threaded, m  =  expected structures threaded and k  =  expected structures in hit list. The p-value threshold for considering a domain as a hit was 0.001.

### Validation through orthologs

Validation through orthologs relied first on the accurate detection of orthologs. This was possible through the use of the Homologene database (where available, [85]) and the Inparanoid 2.0 software [86]. Once orthologs were determined, the orthologous sequences were submitted to the HMMER2 domain search with a higher expect value threshold of 100. If the domain that was found in the original species was also detected in the validation sequence, this region was sent for fold recognition. The scoring procedure for threading was identical to that of the genome-wide runs. If the domain did also positively score in the close ortholog, it was marked as such. The species, score and original hit information were retained for storage in the database.

### Validation through essential, functional residues

Data on functional residues was taken from the CD database. For each HMMerThread weakly conserved domain hit, the corresponding CD alignment was obtained through the use of RPS-BLAST against the CDD [2]. For this alignment, each functional residue was evaluated against the expected functional residue from the CD consensus alignment. Residues were marked as 1) identity if they were the same, 2) similarity, if they had a positive score from comparison in the BLOSUM62 matrix, 3) null, if they do not fall into the first two categories. For the evaluation, a threshold of 25% similarity was used.

### Calculating recall, precision and accuracy of HMMerThread (old and new), GenTHREADER and Superfamily

To evaluate the performance of HMMerThread, two versions of the Pfam database were obtained: Pfam10 (July 2003) and Pfam22 (July 2007). Common conserved domains seen in both versions were extracted resulting in 5248 domains. The resulting HMM databases were calibrated and searched against the human proteome set (RefSeq, September 2007) using hmmpfam. Conserved domains were selected, if they scored < 0.1 in Pfam22, >0.1 in Pfam10 and had an e-value difference of greater than 10 fold. These conserved domains were considered to be True Positives (TPs) in the following analysis. For each conserved domain region, HMMerThread was run with all overlapping domains enabled (up to an e-value of 50) against the Pfam10 profiles. This provided us with ∼1520 potential domains for HMMerThread to distinguish between True Positives (TPs), False Positives (FPs), False Negatives (FNs) and True Negatives (TNs). The True Negative dataset was derived from Pfam10∶22 searches that did not score significantly in either Pfam10 or Pfam22 according to our criteria. Clan members were furthermore excluded from false positive calculations.

Formulas for TPs, FNs, FPs and TNs were as follows:

TP  =  (BestHit ∈ 10∶22PosDS) && (p-value< = 0.001)

FN  =  (BestHit ∈ 10∶22PosDS) && (p-value>0.001)

FP  =  (BestHit ∉ 10∶22PosDS) && (p-value< = 0.001)

TN  =  (BestHit ∉ 10∶22PosDS) && (p-value>0.001),

whereby BestHit is the conserved domain hit discovered as top hit in HMMerThread, 10∶22PosDS are all conserved domains from the Pfam10∶22 dataset qualifying as true positives (see above), and p-value represents the combined probability developed for scoring HMMerThread hits.

Accuracy, recall and precision were determined as follows:

Accuracy  =  (TP+TN)/(TP+TN+FP+FN);

Recall  =  TP/(TP+FN);

Precision  =  TP/(TP+FP).

The old version of HMMerThread was used on the same dataset with standard settings and a hit-depth of 25. The p-value of positively identified conserved domains was set to 0.0000001, negatively identified domains received a p-value of 1. The according p-values were used for calculating TPs, FPs, TNs and FNs. All other procedures were done as described as above.

The local version of Genthreader (pgen 8.2) was used on the Pfam10∶22 remotely conserved domain set. PSIPred 2.5 was used with the uniref90 database for secondary structure prediction. Threading was performed against the SCOP fold library provided from 20th July 2009. In order to score conserved domains, SCOP structures that were scored as “CERTS” were mapped to Pfam domains using the PDBMAP mapping provided by Pfam. These were then compared directly with the Pfam10∶22 list as with HMMerThread (old and new versions). Calculations of TPs, FPs, TNs and FNs followed the above formulas, except that instead of the p-value, the presence (as a CERT domain) or absence of a domain was used as the second criteria. CLAN members were again excluded from false positive calculations.

The local version of Superfamily was downloaded from the Superfamily website and setup according to the instructions on the site using HMMER 2.3.2 as the HMM search program and SCOP 1.73 as the models database. Processing the sequences produced 766 unique superfamily hits, most of which were scored via multiple superfamily models. In order to determine domain level hits, the SCOP IDs from the models that encompassed the hits for each superfamily were used to map to Pfam domains through the PDBMAP mapping provided by Pfam. True positives were scored, if any of the pfam-IDs associated with a superfamily were identified. All other related Pfam families found in the same region were excluded from false positive calculations next to the CLAN members also used in GenThreader and HMMerThread searches. Formulas for calculating TPs, FPs, TNs and FNs followed the above formulas, again using the presence or absence of a domain as the second criteria.

### HMMerThread Database

The HMMerThread Database was implemented in Python 2.4 and MySQL. The web-service is provided by Apache.

### Annotation

Annotation for the database was obtained from species specific sources either from ftp downloads or from HTTP downloads. Annotation in the form of InterPro Domains [87] and CDD domains [2] were obtained by running the stand-alone applications InterProScan [88] and RPS-BLAST [2] against the sequences in the database. Databases used include NCBI [89], SGD [90], Wormbase [91], Flybase [92], PombeDB [93].

### HPRD Overlay

Protein-protein interactions from the HPRD were extracted for each *H. sapiens* protein in the HMMerThread database. For each interaction partner, conserved domains (HMMerThread and Pfam) were matched with known domain-domain interactions from iPfam [76]. If domains in each of the proteins were known to interact, these are displayed as the potential interaction surface that explains the protein-protein interaction in the HPRD.

### Live HMMerThread

The *live* version of HMMerThread was implemented in Python 2.4. The only difference to the Perl implementation is that all of the steps for processing are combined and the handling of web jobs is added. Furthermore, the HTML output capability was added directly in a manner similar to the HMMerThread Database. The HMMerThread *live runner* uses the additional threading module for Python to allow for the submission of jobs simultaneously on different threads. Furthermore, it relies on the SMP capabilities of PSI-BLAST (4 CPUs) and HMMER2 (4 CPUs) along with the MPI implementation in OpenProspect (32 CPUs) to reduce the runtime of the jobs.

### Other bioinformatics methods

PSI-BLAST searches [7] and Phyre runs [26] were carried out using standard settings. hhpred searches [9] were carried out using only orthologs of the analyzed families shown in Figures 4 to 7. Multiple sequence alignments were done using ClustalW [94] and/or Mafft [95] and manually refined. Figures were prepared in Illustrator. Multiple sequence alignments of conserved domain families were taken from NCBI CD-database [2]. For comparison between Pfam22, Pfam24 and the two HMMER releases (HMMER2.3.2 and HMMER3b3), we removed the top 20, promiscuous conserved domains, as well as Zinc Finger domains for the analysis, as HMMerThread has difficulties of identifying the correct family of Zinc Fingers.

## Supporting Information

Figure S1 **Cumulative Distribution Function of threading Z-scores.** When a hypergeometric p-value threshold < 0.05 is used, 90% of the expected conserved domain structures fall within the top 60 positions of threading hits with a Z-score <2.38.
(PDF)

Figure S2 **ROC curve of the performance of the HMMerThread algorithm.** True positives were plotted against false positive predictions of the HMMerThread algorithm. The optimal p-value range corresponds to our chosen cutoff (1E-03), resulting in 14% false positive rate and a 95% true positive rate (see also supplemental Table S3)
(PDF)

Figure S3 **Screenshots of the HMMerThread Database.** (**A**) Overview of one entire record in the HMMerThread database, in this case showing *H. sapiens* APPL1 with all associated annotation. Those include links to original database entries (NCBI), interaction partners, interacting domains and literature including GeneRIFs, Gene Summaries, Gene Ontology information, as well as known sequence-based domains. (**B**) HMMerThread domains image with the validated BAR domain (3 species), displayed by mouse over. The associated results of remotely conserved domains are shown in the HMMerThread hits table and the HMMer alignment of all remotely conserved HMMerThread domains are provided below the hit table.
(PDF)

Table S1 **Species used for cross-species validation of remotely conserved HMMerThread domains.**
(PDF)

Table S2 **Comparison of performance of the old and new version of HMMerThread, GenTHREADER and Superfamily.**
(XLS)

Table S3 **False positive and true positive HMMerThread predictions using different p-value settings.**
(XLS)

Table S4 **Conserved domains (InterProScan, HMMerThread) of hits from a genome-wide screen for cofactors of Hepatitis C Virus replication in human cells**
(XLS)

Table S5 **HMMerThread remotely conserved domains found in DUF domains**
(XLS)

Table S6 **list of known interactors and their interacting domains based on remotely conserved domains**
(XLS)

Table S7 **Accession numbers of sequences used in** **Figures 3** **-** [Fig pone-0017568-g004] [Fig pone-0017568-g005] **6** **.**
(XLS)
