# Supplementary figures and images for: HMMerThread: Detecting Remote, Functional Conserved Domains in Entire Genomes by Combining Relaxed Sequence-Database Searches with Fold Recognition
Source: PLoS One. 2011 Mar 10;6(3):e17568. doi: 10.1371/journal.pone.0017568 (PMC3053371; doi:10.1371/journal.pone.0017568)

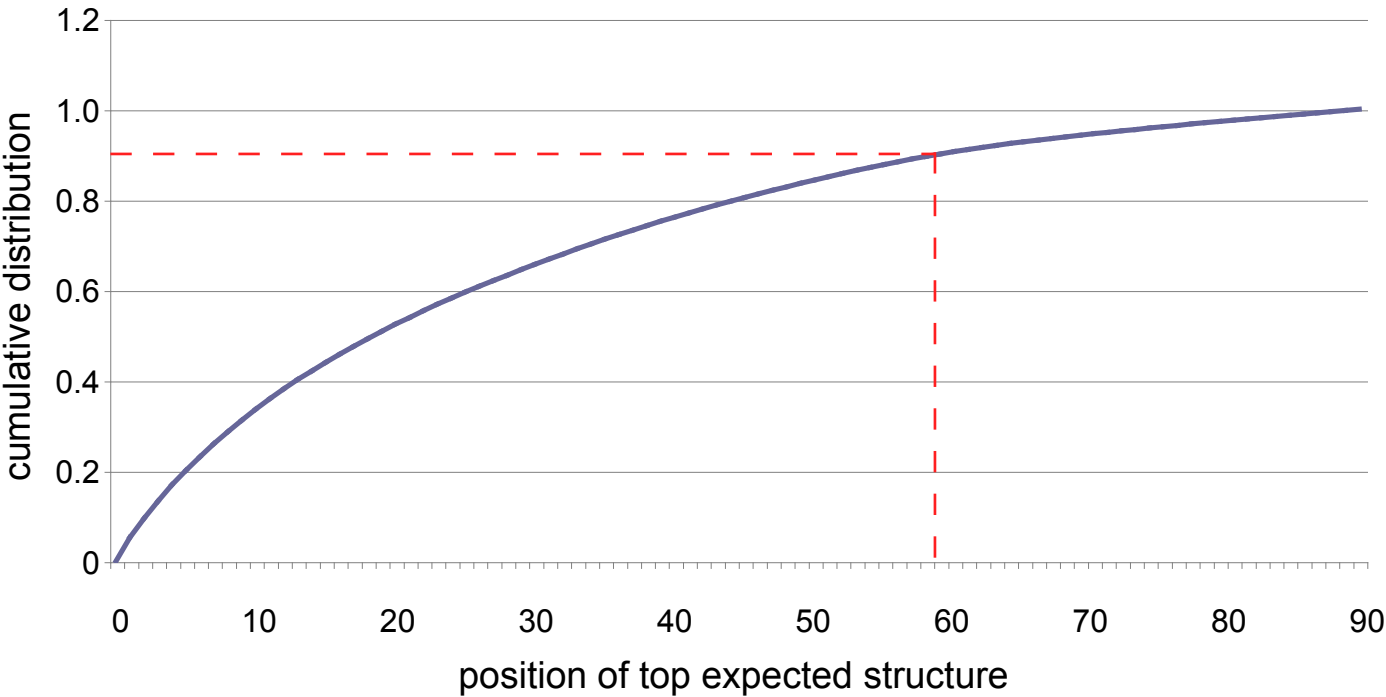

Supplement: Figure S1 — Cumulative Distribution Function of threading Z-scores. When a hypergeometric p-value threshold < 0.05 is used, 90% of the expected conserved domain structures fall within the top 60 positions of threading hits with a Z-score <2.38. (PDF) [file pone.0017568.s001.pdf]

ROC curve of new HMMerThread data

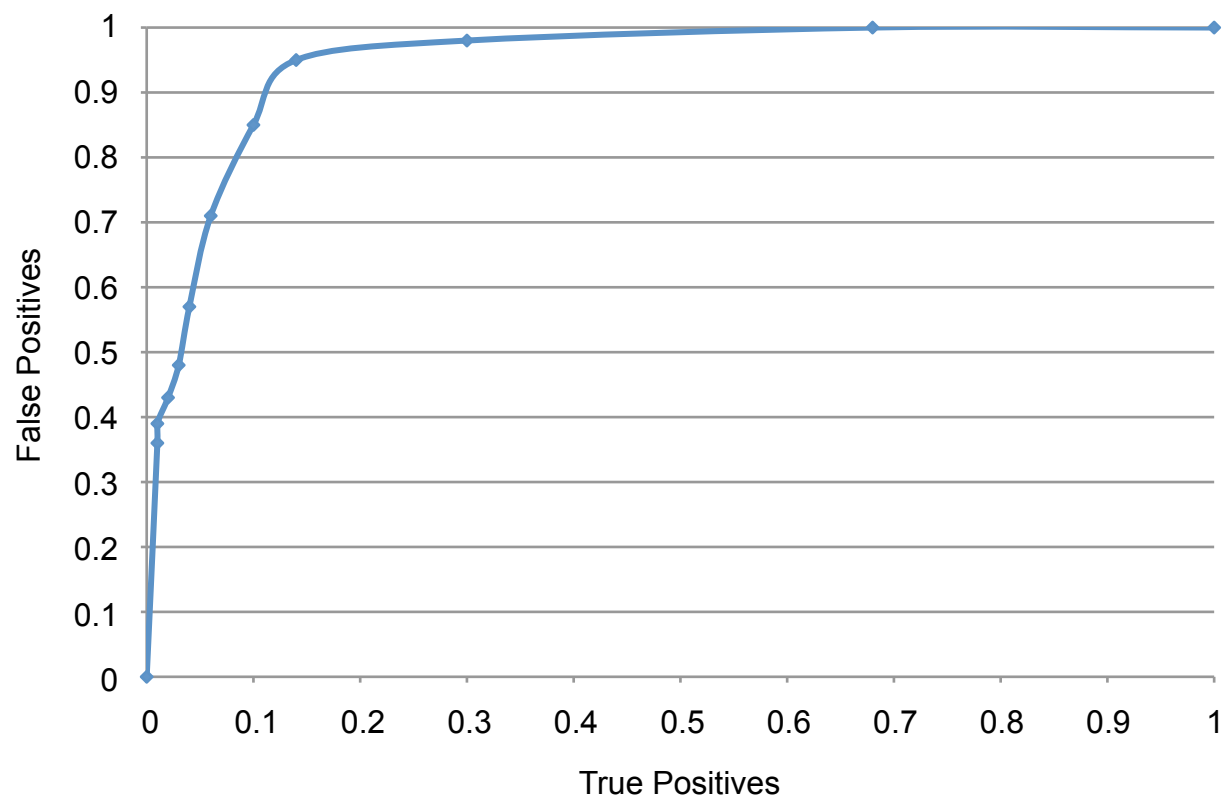

Supplement: Figure S2 — ROC curve of the performance of the HMMerThread algorithm. True positives were plotted against false positive predictions of the HMMerThread algorithm. The optimal p-value range corresponds to our chosen cutoff (1E-03), resulting in 14% false positive rate and a 95% true positive rate (see also supplemental Table S3) (PDF) [file pone.0017568.s002.pdf]

A

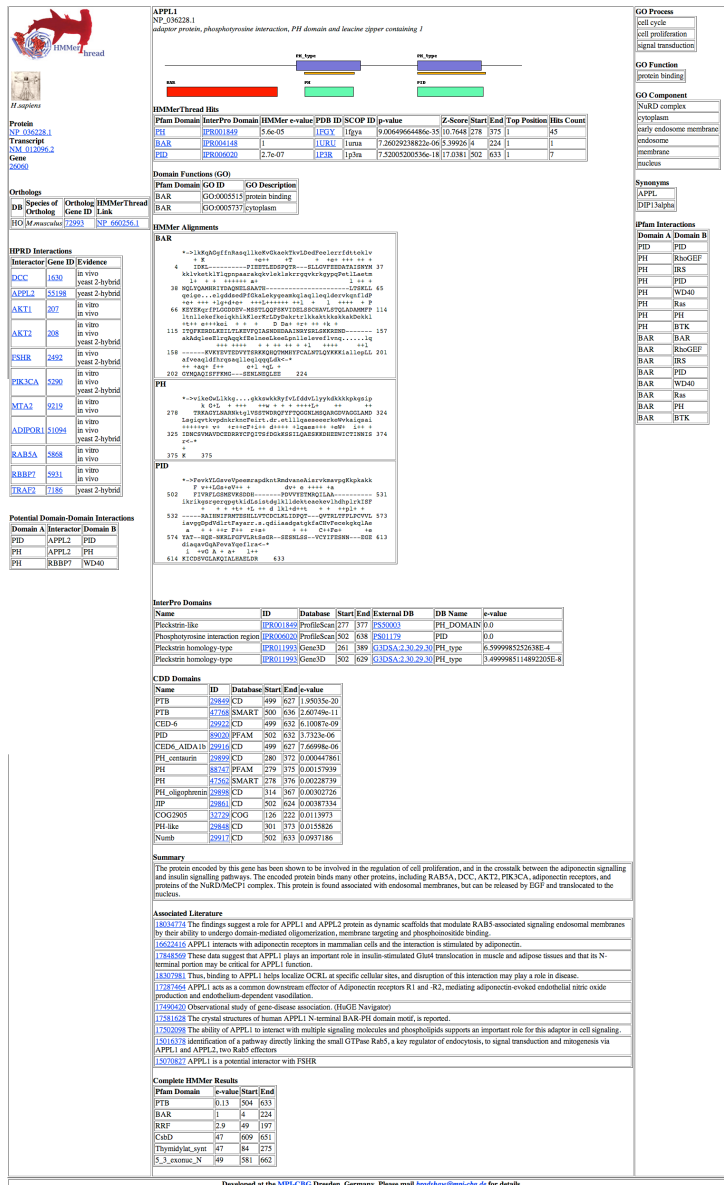

B

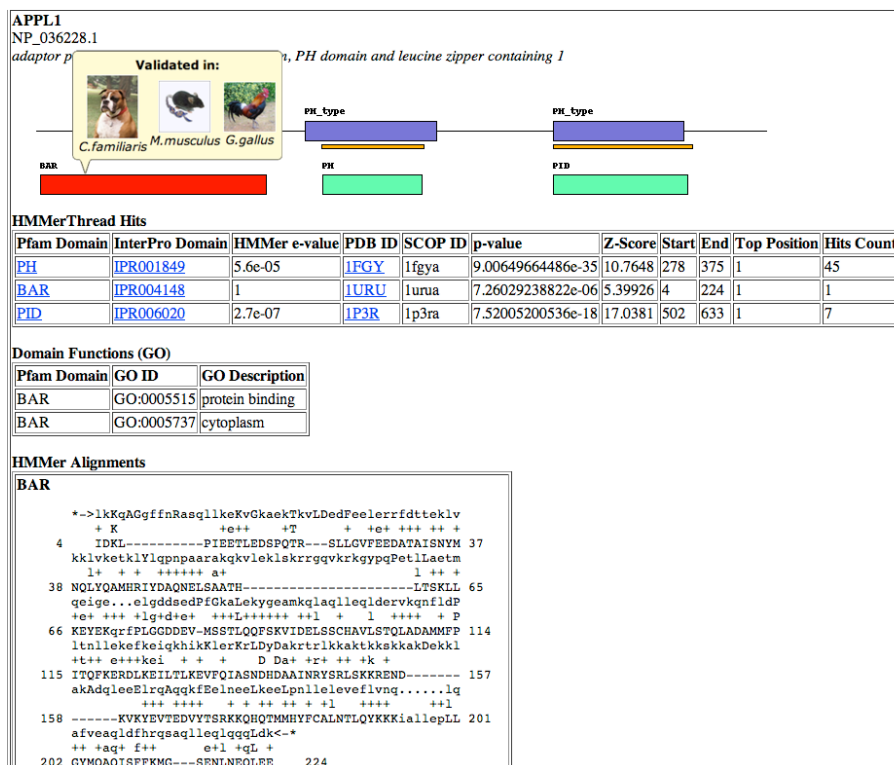

Supplement: Figure S3 — Screenshots of the HMMerThread Database. (A) Overview of one entire record in the HMMerThread database, in this case showing H. sapiens APPL1 with all associated annotation. Those include links to original database entries (NCBI), interaction partners, interacting domains and literature including GeneRIFs, Gene Summaries, Gene Ontology information, as well as known sequence-based domains. (B) HMMerThread domains image with the validated BAR domain (3 species), displayed by mouse over. The associated results of remotely conserved domains are shown in the HMMerThread hits table and the HMMer alignment of all remotely conserved HMMerThread domains are provided below the hit table. (PDF) [file pone.0017568.s003.pdf]
