# Supplementary material for: HMMerThread: Detecting Remote, Functional Conserved Domains in Entire Genomes by Combining Relaxed Sequence-Database Searches with Fold Recognition
Source: PLoS One. 2011 Mar 10;6(3):e17568. doi: 10.1371/journal.pone.0017568 (PMC3053371; doi:10.1371/journal.pone.0017568)
Supplement: Table S1 — Species used for cross-species validation of remotely conserved HMMerThread domains. (PDF) [file pone.0017568.s004.pdf]

Supplemental Table S1: Cross-species validation of remotely conserved domains

**Primary Species**	**Validation Species 1**	**Validation Species 2**	**Validation Species 3**
Homo sapiens	*Canis l. familiaris*	*Mus musculus*	*Gallus gallus*
Mus musculus	*Rattus norvegicus*	*Homo sapiens*	*Gallus gallus*
Danio rerio	*Takifug rubripes*	*Tetraodon nigroviridis*	
Drosophila melanogaster	*Apis mellifera*	*Tribolium castaneum*	*Anopheles gambiae*
Caenorhabditis elegans	*Caenorhabditis briggsae*		
Dictyostelium discoideum			
Schizosaccharomyces pombe			
Saccharomyces cerevisiae	*Kluyveromyces lactis*	*Ashbya gossypii*	*Candida albicans*
